# Transcatheter PFO closure for cryptogenic stroke: current approaches and future considerations

**DOI:** 10.3389/fcvm.2024.1391886

**Published:** 2024-05-20

**Authors:** Moemen Eltelbany, Raghav Gattani, Araba Ofosu-Somuah, Abdulla Damluji, Kelly C. Epps, Wayne B. Batchelor

**Affiliations:** ^1^ Inova Schar Heart and Vascular, Inova Fairfax Medical Campus, Falls Church, VA, United States; ^2^ Cardiovascular Medicine, Brigham and Women’s Hospital, Boston, MA, United States; ^3^ Division of Cardiology, Department of Medicine, University of North Carolina, Chapel Hill, NC, United States

**Keywords:** PFO, cryptogenic stroke, RoPE score, congenital atrial septal defect, PFO closure devices

## Abstract

Patent Foramen Ovale (PFO) is a common congenital atrial septal defect present in 20%–35% of the general population. Although generally considered a benign anatomic variant, a PFO may facilitate passage of a thrombus from the venous to arterial circulation, thereby resulting in cryptogenic stroke or systemic embolization. A PFO is detected in nearly one half of patients presenting with cryptogenic stroke and often considered the most likely etiology when other causes have been excluded. In this review, we discuss the contemporary role of transcatheter closure of PFO in the treatment of cryptogenic stroke, including devices currently available for commercial use in the United States (Amplatzer PFO^TM^ Occluder and Gore^TM^ Cardioform Septal Occluder) and a novel suture-mediated device (NobleStitch^TM^ EL) under clinical investigation. To provide the best care for cryptogenic stroke patients, practitioners should be familiar with the indications for PFO closure and corresponding treatment options.

## Graphical Abstract

PFO closure devices: past present and future. © Devon Medical Art LLC.

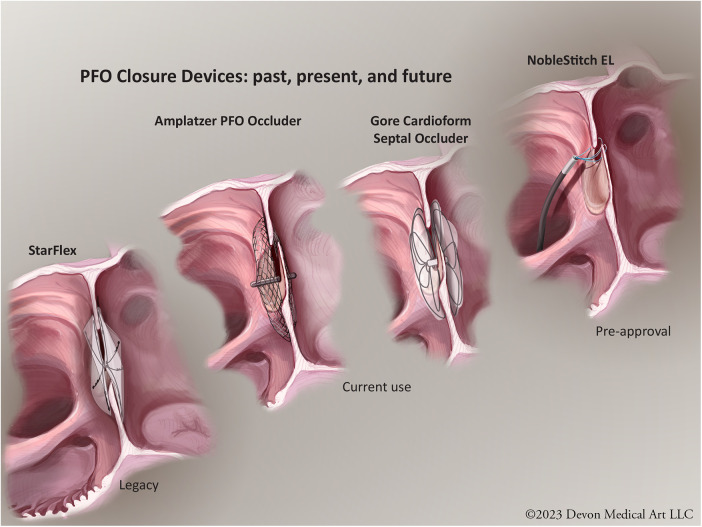

## Introduction

Patent Foramen Ovale (PFO) is a common anatomic variant, present in about 20%–35% of the population (1). During cardiac embryogenesis, the septum primum and the septum secundum form a flap-like valve in the interatrial septum allowing blood to flow from the right to left atrium, bypassing the fetal lungs. After birth, with spontaneous breathing and a reduction of pulmonary vascular resistance, right sided atrial pressure drops compared to left, leading to the fusion and the closure of interatrial septal defect. However, in approximately 1 in 4 individuals, the septa fail to fuse, leaving a PFO which is a residual communication between the right and left atria. While PFOs are generally clinically insignificant, under certain conditions they can allow passage of (1, 2) a thrombus/embolus from the venous system into the arterial system leading to a paradoxical embolism which can result in cryptogenic stroke and/or systemic embolism (3). Transcatheter PFO closure has been developed and performed for decades in patients with presumed paradoxical embolism to reduce the risk of recurrent cardio-embolic events.

### Indications for PFO closure with cryptogenic stroke

Stroke is the fifth leading cause of death in the US and a major contributor to cardiovascular morbidity and mortality. Cryptogenic strokes represent between 15% and 40% of all strokes (4). The Trial of Org 10172 in Acute Stroke Treatment (TOAST) defined cryptogenic stroke as a brain infarct not related to a definite source of cardio embolism, large vessel occlusion or small vessel disease despite extensive evaluation (5). Prior retrospective studies have revealed an increased prevalence of PFO (40%–45%) in patients with cryptogenic stroke (3, 6). However, given the high prevalence of PFO in the general population, when selecting patients for PFO closure, it is important to determine the likelihood that the PFO is causally implicated in the stroke rather than being an innocent bystander. The Risk Of Paradoxical Embolism score (RoPE) score is a 10-point scoring scale developed to predict the likelihood that a cryptogenic stroke is attributable to an associated PFO, rather than an incidental finding (Table 1). As a validated tool for objectively assessing the relationship between cryptogenic stroke and PFO in a given patient, the RoPE score helps guide PFO closure treatment decisions (7, 8), keeping in mind that the estimation of degree of causality of PFO in cryptogenic stroke using the RoPE score may be affected by the difference in PFO prevalence in population (9). While no significant race-ethnic differences in PFO prevalence were identified in a large US cohort of cryptogenic stroke patients, white and Hispanic patients had a higher prevalence of high risk PFO anatomic features compared to black patients (10). In addition to clinical variables accounted for in the RoPE score, anatomic characteristics of the PFO are also now recognized as important features in determining PFO related stroke risk. The PFO-Associated Stroke Causal Likelihood (PASCAL) Classification System adds echocardiographic features, such as PFO shunt size and presence of interatrial septal aneurysm, to the RoPE score to help further risk stratify patients into those who benefit most from PFO closure (11).

**Table 1:** RoPE score calculator.

Characteristic	Points	RoPE score
No history of hypertension	1	
No history of diabetes	1	
No history of stroke of TIA	1	
Nonsmoker	1	
Cortical infarct on imaging	1	
Age year		
18–29	5	
30–39	4	
40–49	3	
50–59	2	
60–69	1	
≥70	0	
Total score (sum of individual points)		
Maximum score (a patient <30 year with no hypertension, no diabetes, no history of stroke or TIA, nonsmoker, and cortical infarct)		10
Minimum score (a patient ≥70 year with hypertension, diabetes, prior stroke, current smoker and no cortical infarct)		0

RoPE, risk of paradoxical embolism.

The debate about whether to close a PFO to reduce the risk of recurrent stroke or recurrent paradoxical embolism has been ongoing over the last two decades. Early clinical trials, including the Evaluation of the STARFlex Closure System in Patients with a Stroke and/or Transient Ischemic Attack due to Presumed Paradoxical Embolism Through a Patent Foramen Ovale [CLOSURE I] and the Percutaneous Closure of Patent Foramen Ovale Using the Amplatzer PFO Occluder With Medical Treatment in Patients With Cryptogenic Embolism [PC Trial] failed to show significant benefit of PFO closure in patients with cryptogenic stroke or TIA (12, 13). However, these trials were hampered by several design issues, including limited statistical power, a high crossover rate between the study groups, patient selection issues, and lack of standardization of antithrombotic therapy in the medical therapy group. Additionally, the STARFlex device used in CLOSURE I trial was withdrawn in Europe out of concerns about residual defects and device-related thrombus formation (14, 15).

Subsequent innovation led to improved closure device and study designs. The Randomized Evaluation of Recurrent Stroke Comparing PFO Closure to Established Current Standard of Care Treatment (RESPECT) trial initially failed to show superiority of PFO closure over medical therapy, however after extended follow up (median 5.9 years) a significant reduction in the incidence of ischemic stroke compared to medical therapy was observed [HR 0.55; 95% CI (0.31–0.999); *p* = 0.046] (16, 17). Several other randomized control trials, designed to address the pitfalls of earlier trials, further validated the benefit of PFO closure compared to medical therapy in reducing the risk of recurrent stroke in patients presenting with cryptogenic stroke. The Gore Septal Occluder Device for PFO Closure in Stroke Patients (REDUCE) trial showed remarkable benefit and reduction in the risk of ischemic stroke compared to medical therapy alone (1.4 vs. 5.5%; *p* = 0.002; NNT = 25) (18). In the Patent Foramen Ovale Closure or Anticoagulation vs. Antiplatelet after Stroke (CLOSE) trial, none of the 238 patient who were randomized for PFO closure had stroke compared to 14 strokes in the medical treatment arm (hazard ratio, 0.03; 95% confidence interval, 0–0.26; *P* < 0.001) (19). In the Device Closure Versus Medical Therapy for Cryptogenic Stroke Patients with High-Risk PFO (DEFENSE-PFO trial) PFO closure significantly reduced the composite of stroke, vascular death, and thrombolysis in MI major bleeding at 2 years compared to medical therapy with number needed to treat (NNT) of 8 (20). Mojadidi et al., conducted a meta-analysis including the CLOSE, RESPECT, REDUCE, PC and CLOSURE trials (*n* = 3,440). This confirmed that percutaneous PFO closure reduced the risk of ischemic stroke compared to medical therapy alone (2.0% vs. 4.5%; RR: 0.42; 95% CI: 0.20–0.91; *p* = 0.027). However, the risk of atrial fibrillation was noted to be higher in the device arms (4.2% vs. 0.74%; RR: 4.55; 95% CI: 2.16–9.60; *p* < 0.0001), which was likely procedure related (21).

Based on this robust clinical trial evidence base, the Society for Cardiovascular Angiography and Interventions recently released guidelines for the management of PFO (22). The guidelines gave its strongest recommendations for PFO closure over antithrombotic therapy in patients between ages 18 and 60 years with a PFO-associated stroke. Similarly, American Heart Association/American Stroke Assocation Guideline and the Canadian Stroke Best Practices Recommendations gave the same recommendation for PFO closure in patients within the same age group with cryptogenic stroke; however, both of these guidelines also emphasize the importance of anatomic features by recommending closure specifically in patients with high risk PFO features (23, 24). ESC guidelines also recommend PFO closure in patients with PFO-associated stroke and have extended the recommendation for PFO closure to include patients up to 65 years old (25). When considering applications of these guidelines to individual patients, it is important to note that a RoPE score ≥7 has been associated with greater benefit from PFO closure. An analysis of data from CLOSURE-1, RESPECT and PC trials demonstrated that in patient with RoPE score of 7 and more, the rate of recurrent strokes per 100 person-years was 0.30 in the PFO closure group vs. 1.03 in the medical therapy group [hazard ratio (HR), 0.31; 95% CI: 0.11–0.85; *P* = 0.02]. In patients with lower RoPE scores (<7, *n* = 912), the rate of recurrent strokes per 100 person-years was 1.37 in the PFO closure group vs. 1.68 in the medical therapy group (HR, 0.82; 95% CI: 0.42–1.59; *P* = 0.56) (26).

### Transcatheter PFO closure device therapy

The choice of a PFO closure device depends on several factors, including patient anatomy, size of the PFO, physician preference and experience, and the safety and efficacy profile of the device (27). There are currently two FDA approved PFO closure devices, based on results from the RESPECT and REDUCE trials. The Amplatzer^TM^ PFO Occluder is a double-disc nitinol wire mesh device that conforms to the septal wall and is delivered via a catheter through the vein under echocardiographic and fluoroscopic guidance. The discs are deployed on either side of the atrial septum, effectively sealing the PFO. The device comes in 4 sizes (18, 25, 30, and 35 mm), accommodating varying PFO anatomy (16, 17, 23). The Gore^TM^ Cardioform Septal Occluder, manufactured by W.L. Gore & Associates, also uses a dual-disc design, and is made of a nickel-titanium alloy frame covered with a soft, flexible material composed of expanded polytetrafluoroethylene (ePTFE) -a biocompatible material that conforms to the septum. The device comes in 3 sizes (20, 25, and 30 mm), and has a lower profile which may reduce the risk of cardiac tissue irritation (23, 28). Both the Amplatzer and the Gore devices proved to be superior to STARFlex device in terms of reducing the risk of recurrent stroke/TIA with less occurrence of new onset Afib (29). Occlutech® Flex II PFOOccluder is a newer device currently under investigation in the FDA approved OCCLUFLEX study.OCCLUFLEX is a prospective randomized multi-center controlled investigation comparing PFO outcomes of the Occlutech Flex II Occluder to standard of care PFO occlusion with the Amplatzer PFO occluder and the Gore Cardioform Septal Occluder. Using nitinol wire mesh, the Occlutech device consists of 2 retention discs connected by a 3 mm central waist with a single hub to promote endothelialization (30, 31). The OCCLUFLEX study is expected to be completed in 2026.

Before considering transcatheter PFO closure, the indications, risks, benefits, and alternatives should be thoroughly discussed, and patient shared decision-making utilized. The currently approved transcatheter PFO closure devices are delivered from 8 to 12 Fr femoral venous sheaths using intracardiac (ICE) or transesophageal echocardiography (TEE) for imaging guidance during the procedure. Intracardiac echocardiography is often preferred as it facilitates conscious sedation, quicker recovery and earlier hospital discharge (often same-day discharge) than TEE. Postprocedural care is a critical component of the procedure that is essential to effective and safe PFO closure. Oral dual antiplatelet therapy with aspirin and a P2Y12 inhibitor is generally recommended for 6 months to minimize the risk of device thrombus (32) while complete endothelialization occurs over the ensuing several months (17, 33). In some cases, anticoagulants may be prescribed instead of antiplatelet therapy, particularly if there is a high thrombotic risk or the patient has another indication for anticoagulation (23). Pre and post procedural patient education is paramount and should include informing the patient of the signs and symptoms of complications, such as infection, arrhythmia, pericardial effusion or thromboembolism. Transthoracic Echocardiography with agitated saline bubble study is then typically performed at 1-, 6- and 12-months post closure to confirm PFO closure. Successful closure is defined as a grade 0 or 1 residual right to left shunt (34, 35). Although the risk of infective endocarditis after PFO closure is low, prophylactic antibiotics are generally recommended for procedures that could introduce bacteria into the bloodstream during the first 6 months after device implantation (23).

### Transcatheter suture-mediated PFO closure

Despite the excellent efficacy and safety of traditional PFO closure devices, the occasional induction of rhythm disturbances (usually atrial fibrillation), nickel allergy, and rare occurrence of potentially serious complications (device dislodgement, fracture, embolization, infection, and thrombosis) have served as an impetus to consider alternative catheter-based suture-mediated techniques to achieve PFO closure. Furthermore, the permanence of PFO closure device across the interatrial septum may hinder future cardiac interventions that require transseptal access, such as left atrial appendage closure, arrythmia ablations, and transcatheter mitral valve interventions. The NobleStitch® EL Patent Foramen Ovale (PFO) closure device (NobleStitch EL, HeartStitch, Inc., Fountain Valley, CA, USA) is a suture mediated “deviceless” closure system, composed of polypropylene sutures. There are 2 suture delivery catheters, one to capture the septum secundum and one to capture the septum primum. A third Kwiknot catheter is then used to tie the two sutures together, effectively closing the PFO and potentially mitigating the risks and/or limitations of a permanently implanted cardiac device. NobleStitch EL has received CE Mark in the European Union and 510k approval by the FDA for the broad indication of cardiovascular suturing, but not specifically for the indication of PFO closure for secondary stroke prevention.

Prospective observational studies have provided data on the feasibility, safety, and efficacy of the NobleStitch® EL PFO closure system, however, thus far there have been no studies comparing results directly to commercially available PFO closure devices. The NobleStitch® EL Italian registry included 200 consecutively enrolled patients from June 2016 to October 2017 (mean age 44 +/–13 years, 59% female) and demonstrated successful PFO closure with the NobleStitch EL in 96% of the patients, with closure rates comparable to traditional device-based systems. Intermediate term follow-up with agitated saline contrast echocardiography with valsalva maneuver revealed no (grade 0) right to left shunt (RLS) in 75% of patients, RLS grade ≤1 in 89% and significant residual RLS (grade 2 and 3) was present in 11%. Using the standard definition of effective closure (grade 0 or 1 residual RLS), NobleStitch® EL system achieved closure rates similar to that previously reported for established closure devices and long-term follow-up data at 2 years has shown this approach to be safe and effective with thus far no major long-term complications, or permanent arrhythmias (36).

There are several important anatomical features to consider when performing PFO closure using the NobleStitch EL device. A recent retrospective observational study showed that a PFO width of >5 mm and larger (grade 2–3) preprocedural right to left shunts were associated with a lower likelihood of successful PFO closure with 1 suture (37). Utilization of more than one stitch for complete closure in these cases may be necessary. Long-tunnel shaped PFO and septal aneurysms can sometimes be challenging for successful closure using NobleStitch (38–40). Another prospective single center study with a 6-month follow-up revealed partial stitch detachment, atrial septal tear and knot embolization as the main causes of RLS at follow up (41, 42). Therefore, despite some theoretical advantages and early enthusiasm with Noblestich EL, it remains unclear if this approach will provide as effective and complete PFO closure as the 2 established FDA-approved devices. The ongoing NobleStitch EL STITCH Trial (NCT04339699), a prospective, non-randomized, open-label study comparing NobleStitch with the Amplatzer PFO Occluder, should provide further information on the relative efficacy of these 2 devices, including PFO closure success rates, reduction of recurrent ischemic events and safety.

## Conclusions

Although PFO is quite common in the general population, this congenital atrial septal defect occasionally results in cryptogenic stroke or paradoxical systemic embolism. The totality of clinical trial evidence argues in favor of PFO closure over medical therapy alone in patients presenting with cryptogenic stroke, especially those between ages 18 and 60 years with high RoPE scores, large shunts and/or atrial septal aneurysms. There are currently 2 FDA-approved PFO closure devices available for use in the U.S. that have demonstrated safety and efficacy. Although registry data also appears promising for suture mediated PFO closure, the relative efficacy and safety of this approach vs. established PFO closure devices has yet to be established, thereby warranting further investigation. To provide the best care for cryptogenic stroke patients, practitioners should be familiar with the indications for PFO closure in this setting and corresponding treatment options.
